# Barriers to Provider-Initiated Testing and Counselling for Children in a High HIV Prevalence Setting: A Mixed Methods Study

**DOI:** 10.1371/journal.pmed.1001649

**Published:** 2014-05-27

**Authors:** Katharina Kranzer, Jamilah Meghji, Tsitsi Bandason, Ethel Dauya, Stanley Mungofa, Joanna Busza, Karin Hatzold, Khameer Kidia, Hilda Mujuru, Rashida A. Ferrand

**Affiliations:** 1 Department of Infectious Disease Epidemiology, London School of Hygiene & Tropical Medicine, London, United Kingdom; 2 Biomedical Research and Training Institute, Harare, Zimbabwe; 3 Harare City Health Department, Harare, Zimbabwe; 4 Department of Population Health, London School of Hygiene & Tropical Medicine, London, United Kingdom; 5 Population Services International, Harare, Zimbabwe; 6 Department of Paediatrics, University of Zimbabwe, Harare, Zimbabwe; 7 Clinical Research Department, London School of Hygiene & Tropical Medicine, London, United Kingdom; National Institute of Child Health and Human Development, United States of America

## Abstract

**Background:**

There is a substantial burden of HIV infection among older children in sub-Saharan Africa, the majority of whom are diagnosed after presentation with advanced disease. We investigated the provision and uptake of provider-initiated HIV testing and counselling (PITC) among children in primary health care facilities, and explored health care worker (HCW) perspectives on providing HIV testing to children.

**Methods and Findings:**

Children aged 6 to 15 y attending six primary care clinics in Harare, Zimbabwe, were offered PITC, with guardian consent and child assent. The reasons why testing did not occur in eligible children were recorded, and factors associated with HCWs offering and children/guardians refusing HIV testing were investigated using multivariable logistic regression. Semi-structured interviews were conducted with clinic nurses and counsellors to explore these factors. Among 2,831 eligible children, 2,151 (76%) were offered PITC, of whom 1,534 (54.2%) consented to HIV testing. The main reasons HCWs gave for not offering PITC were the perceived unsuitability of the accompanying guardian to provide consent for HIV testing on behalf of the child and lack of availability of staff or HIV testing kits. Children who were asymptomatic, older, or attending with a male or a younger guardian had significantly lower odds of being offered HIV testing. Male guardians were less likely to consent to their child being tested. 82 (5.3%) children tested HIV-positive, with 95% linking to care. Of the 940 guardians who tested with the child, 186 (19.8%) were HIV-positive.

**Conclusions:**

The HIV prevalence among children tested was high, highlighting the need for PITC. For PITC to be successfully implemented, clear legislation about consent and guardianship needs to be developed, and structural issues addressed. HCWs require training on counselling children and guardians, particularly male guardians, who are less likely to engage with health care services. Increased awareness of the risk of HIV infection in asymptomatic older children is needed.

*Please see later in the article for the Editors' Summary*

## Abstract 2

Rashida Ferrand and colleagues combine quantitative and qualitative methods to investigate HIV prevalence among older children receiving primary care in Harare, Zimbabwe, and reasons why providers did not pursue testing.

*Please see later in the article for the Editors' Summary*

## Editors' Summary

**Background:**

Over 3 million children globally are estimated to be living with HIV (the virus that causes AIDS). While HIV infection is most commonly spread through unprotected sex with an infected person, most HIV infections among children are the result of mother-to-child HIV transmission during pregnancy, delivery, or breastfeeding. Mother-to-child transmission can be prevented by administering antiretroviral therapy to mothers with HIV during pregnancy, delivery, and breast feeding, and to their newborn babies. According to a report by the Joint United Nations Programme on HIV/AIDS published in 2012, 92% of pregnant women with HIV were living in sub-Saharan Africa and just under 60% were receiving antiretroviral therapy. Consequently, sub-Saharan Africa is the region where most children infected with HIV live.

**Why Was This Study Done?:**

If an opportunity to prevent mother-to-child transmission around the time of birth is missed, diagnosis of HIV infection in a child or adolescent is likely to depend on HIV testing in health care facilities. Health care provider–initiated HIV testing and counselling (PITC) for children is important in areas where HIV infection is common because earlier diagnosis allows children to benefit from care that can prevent the development of advanced HIV disease. Even if a child or adolescent appears to be in good health, access to care and antiretroviral therapy provides a health benefit to the individual over the long term. The administration of HIV testing (and counselling) to children relies not only on health care workers (HCWs) offering HIV testing but also on parents or guardians consenting for a child to be tested. However, more than 30% of children in countries with severe HIV epidemics are AIDS orphans, and economic conditions in these countries cause many adults to migrate for work, leaving children under the care of extended families. This study aimed to investigate the reasons for acceptance and rejection of PITC in primary health care settings in Harare, Zimbabwe. By exploring HCW perspectives on providing HIV testing to children and adolescents, the study also sought to gain insight into factors that could be hindering implementation of testing procedures.

**What Did the Researchers Do and Find?:**

The researchers identified all children aged 6 to 15 years old at six primary care clinics in Harare, who were offered HIV testing as part of routine care between 22 January and 31 May 2013. Study fieldworkers collected data on numbers of child attendances, numbers offered testing, numbers who underwent HIV testing, and reasons why HIV testing did not occur. During the study 2,831 children attending the health clinics were eligible for PITC, and just over half (1,534, 54.2%) underwent HIV testing. Eighty-two children tested HIV-positive, and nearly all of them received counselling, medication, and follow-up care. HCWs offered the test to around 75% of those eligible. The most frequent explanation given by HCWs for a diagnostic test not being offered was that the child was accompanied by a guardian not appropriate for providing consent (401 occasions, 59%); Other reasons given were a lack of available counsellors or test kits and counsellors refusing to conduct the test. The likelihood of being offered the test was lower for children not exhibiting symptoms (such as persistent skin problems), older children, or those attending with a male or a younger guardian. In addition, over 100 guardians or parents provided consent but left before the child could be tested.

The researchers also conducted semi-structured interviews with 12 clinic nurses and counsellors (two from each clinic) to explore challenges to implementation of PITC. The researchers recorded the factors associated with testing not taking place, either when offered to eligible children or when HCWs declined to offer the test. The interviewees identified the frequent absence or unavailability of parents or legal guardians as an obstacle, and showed uncertainty or misconceptions around whether testing of the guardian was mandatory (versus recommended) and whether specifically a parent (if one was living) must provide consent. The interviews also revealed HCW concerns about the availability of adequate counselling and child services, and fears that a child might experience maltreatment if he or she tested positive. HCWs also noted long waiting times and test kits being out of stock as practical hindrances to testing.

**What Do These Findings Mean?:**

Prevalence of HIV was high among the children tested, validating the need for PITC in sub-Saharan health care settings. Although 76% of eligible attendees were offered testing, the authors note that this is likely higher than in routine settings because the researchers were actively recording reasons for not offering testing and counselling, which may have encouraged heath care staff to offer PITC more often than usual. The researchers outline strategies that may improve PITC rates and testing acceptance for Zimbabwe and other sub-Saharan settings. These strategies include developing clear laws and guidance concerning guardianship and proxy consent when testing older children for HIV, training HCWs around these policies, strengthening legislation to address discrimination, and increasing public awareness about HIV infection in older children.

**Additional Information:**

Please access these websites via the online version of this summary at http://dx.doi.org/10.1371/journal.pmed.1001649.

- This study is further discussed in a *PLOS Medicine* Perspective by Davies and Kalk
- The Joint United Nations Programme on HIV/AIDS publishes an annual report on the global AIDS epidemic, which provides information on progress towards eliminating new HIV infections
- The World Health Organization has more information on mother-to-child transmission of HIV
- The World Health Organization's website also has information about treatment for children living with HIV
- Personal stories about living with HIV/AIDS, including stories from young people infected with HIV, are available through Avert, through NAM/aidsmap, and through the charity website Healthtalkonline

## Introduction

Thirty years after the advent of the HIV pandemic, more than 3 million children globally are living with HIV, 90% of them in sub-Saharan Africa [1]. Although numbers of infant infections have fallen by 40% in the last decade because of scale-up of interventions to prevent mother-to-child transmission, global coverage of such programmes remains suboptimal: an estimated 1,000 infant infections occurred daily in 2011 [1]. In addition, coverage of early infant diagnosis among HIV-exposed infants is highly variable, ranging from 10% to 80%—with nearly half of the priority countries having a coverage of under 20%—and only approximately 15% of HIV-infected infants have access to antiretroviral therapy (ART) following diagnosis [2],[3]. For those not diagnosed in infancy, subsequent diagnosis largely depends on HIV testing in health care facilities. We have previously described the substantial burden of undiagnosed HIV in older children and adolescents, the majority of whom are diagnosed only after presentation with advanced disease [4]–[8]. The coverage of ART among children significantly lags behind that in adults (34% in children versus 68% in adults in 2012), and strategies to enable diagnosis and prompt linkage to care for HIV-infected children are crucial to close this gap [9].

HIV testing of children is complex and relies not only on health care workers (HCWs) offering HIV testing but also on guardians consenting for their child to be tested. The extent to which HIV testing for children is implemented at health care facilities is not routinely reported [10],[11]. This study aims to investigate the provision and uptake of provider-initiated HIV testing and counselling (PITC) among children in primary health care settings, and to explore HCW perspectives on provision of HIV testing to children.

## Methods

### Ethical Considerations

HIV testing was carried out with guardian consent and child assent for clients aged under 16 y. Emancipated minors gave independent consent. Written informed consent was obtained from HCWs prior to interviews. Ethical approval for the study was obtained from the Medical Research Council of Zimbabwe and the ethics committees of the Harare City Health Department, the Biomedical Research and Training Institute, and the London School of Hygiene & Tropical Medicine.

### Quantitative Methods

#### Study participants

All children aged 6 to 15 y attending six primary health care clinics in Harare, Zimbabwe, for acute care between 22 January and 31 May 2013 were offered HIV testing as part of routine care by primary care nurses. Study fieldworkers prospectively collected data on numbers of child attendances, numbers offered testing, numbers who underwent HIV testing, and reasons why HIV testing did not occur. This age group was selected because individuals who are aged 16 y and older are able to consent to HIV testing themselves, and children below 6 y are able to access HIV testing through Mother and Child Health services. Criteria for not offering HIV testing were a documented HIV test result in the past 6 mo, known HIV-positive serostatus, attending without a guardian (unless an emancipated minor), or being seriously unwell (requiring immediate hospitalisation or moribund).

#### Study design and intervention

According to Zimbabwean law, children under 16 y require consent from a guardian to undergo HIV testing [12]. Unless the child or guardian declined, HIV testing was carried out by the primary care counsellor following national guidelines using a rapid HIV test kit (Abbott Determine). All positive test results were confirmed with another test kit (SD Bioline), and discordant test results were resolved using a third tie-breaker test (INSTI). The HIV test result was available within an hour of testing. From April 2013 onwards, as a result of supply chain issues, the testing kits were changed so that First Response was used as the first-line test and Abbott Determine was used as the confirmatory test. HIV testing was also offered to the accompanying guardian of any child testing HIV-positive, and s/he was counselled about the importance of the parents and natural siblings of the newly diagnosed child also undergoing HIV testing.

Decentralised HIV care, including initiation of ART, was introduced at the study clinics to facilitate linkage to HIV care, and children who tested HIV-positive were referred for HIV care at the same clinic where they had undergone HIV testing. Demographic details and brief clinical history (children only) of clinic attendees and the accompanying caregivers, and reasons why HIV testing did not occur in eligible children, were recorded.

#### Data analysis

Data were analysed using STATA version 12.0 (StataCorp). Categorical variables were compared using the Chi-squared test or *t*-test, as appropriate. Univariate analysis was used to investigate patient and guardian characteristics associated with HCWs offering and children/guardians refusing HIV testing. Child characteristics included age, gender, orphanhood status, history of hospital admissions, health status in the past 3 mo, and persistent skin complaints. Guardian characteristics included age, gender, and HIV status. Risk factors significant at the *p* = 0.1 level in the univariate analysis were included in multivariable logistic regression models. Records with missing data were not included in the analysis.

### Qualitative Methods

Following analysis of HIV testing data, semi-structured interviews were conducted with HCWs to explore reasons for why testing did not occur in some eligible children. Two trained female social scientists, educated to master's degree level and with extensive experience in conducting HIV-related qualitative research, conducted the interviews. Although unconnected to provision of PITC, both fieldworkers regularly work with the national HIV programme and are thus familiar with the intervention aims and activities. A topic guide was used to interview respondents about their observations and experiences of PITC provision, responses of clients to PITC, characteristics of family members who were more or less likely to consent to testing, and providers' own recommendations for improving PITC uptake.

A total of 12 HCWs were recruited, two from each of the six study clinics. Ten were female and two male. The head nurse and the clinic counsellor were selected to capture the perspectives of both supervisory and frontline staff involved in PITC; all approached staff members agreed to participate. Interviews took place in a private room at each clinic and were conducted in English. Interviews lasted approximately 30 to 45 min and were audio-recorded and subsequently transcribed by the two fieldworkers.

Two authors (K. K. and J. B.) independently analysed the data using basic content analysis. Each coded the transcripts using a framework that included predetermined parent codes based on the interview topic guides (e.g., attitudes to provider-initiated testing, perceptions of caregivers' reasons for refusing testing) and subsequently added emerging themes (e.g., staff uncertainty about policies and procedures, infrastructure weaknesses). The authors refined sub-codes and reconciled differences through face-to-face meetings and e-mail correspondence with each other.

## Results

### Characteristics of the Study Population

There were 3,994 primary care clinic visits by children aged 6–15 y between 22 January and 31 May 2013. Of these, 130 (3.3%) children had evidence of being tested for HIV in the 6 mo prior to the clinic visit, 376 (9.4%) were already known to be HIV-infected, and 657 (16.4%) attended alone or with an unrelated adult (domestic worker, lodger, or neighbour), leaving 2,831 (71.0%) children eligible for PITC. The median age of children eligible for PITC was 9 y (interquartile range [IQR]: 7–11 y), and 53.2% were male (Table 1). 297 (14.3%) were single or double orphans, and 290 (9.5%) had been admitted to hospital previously. Recurrent skin problems and recent poor health were reported by 352 (12.8%) and 192 (7.0%), respectively. The majority of children were accompanied by female guardians.

**Table 1:** Association between baseline variables and not being offered HIV testing by clinic providers.

Category	Variable	All Children (*n* = 2,831)	PITC Not Offered (*n* = 680)	PITC Offered (*n* = 2,151)	Univariate OR (95% CI)	Multivariable OR^a^(95% CI)
**Child**	**Age in years, median (IQR)**	9 (7–11)	9 (8–11)	9 (7–11)		
	**Age by category, ** ***n*** ** (percent)**					
	≤7 y	766 (27.1%)	158 (23.2%)	608 (28.3%)	1	1
	8–11 y	1,387 (49.0%)	342 (50.3%)	1,045 (48.6%)	1.26 (1.02–1.56)	1.23 (0.98–1.55)
	>11 y	678 (24.0%)	180 (26.5%)	498 (23.2%)	1.39 (1.09–1.78)	1.32 (1.01–1.73)
	**Male, ** ***n*** ** (percent)** b	1,505 (53.2%)	338 (49.7%)	1,167 (54.3%)	0.83 (0.70–0.99)	0.82 (0.68–0.99)
	**Orphan, ** ***n*** ** (percent)** c	384 (14.0%)	87 (13.1%)	297 (14.3%)	0.90 (0.70–1.17)	—
	**Previous hospital admission, ** ***n*** ** (percent)** d	260 (9.5%)	47 (7.1%)	213 (10.2%)	0.67 (0.48–0.93)	0.75 (0.53–1.06)
	**Poor health, ** ***n*** ** (percent)** e	192 (7.0%)	37 (5.6%)	155 (7.5%)	0.73 (0.51–1.06)	0.70 (0.47–1.05)
	**Skin problems, ** ***n*** ** (percent)** f	352 (12.8%)	72 (10.8%)	280 (13.5%)	0.78 (0.59–1.02)	0.82 (0.61–1.10)
**Guardian**	**Age in years, median (IQR)** g	33 (28–40)	30 (22.5–39)	34 (29–40)		
	**Age by category, ** ***n*** ** (percent)**					
	≤25 y	435 (15.4%)	229 (33.7%)	206 (9.6%)	4.85 (3.83–6.14)	4.58 (3.58–5.86)
	26–35 y	1,233 (43.7%)	230 (33.8%)	1,003 (46.8%)	1	1
	36–40 y	478 (16.9%)	72 (10.6%)	406 (18.9%)	0.77 (0.60–1.03)	0.72 (0.54–0.97)
	>40 y	678 (24.6%)	149 (21.9%)	529 (24.7%)	1.23 (0.97–1.55)	1.13 (0.89–1.44)
	**Male, ** ***n*** ** (percent)** h	441 (15.7%)	146 (21.6%)	295 (13.8%)	1.72 (1.38–2.14)	1.45 (1.14–1.85)

a *n* = 2,718 for the multivariable model. All variables significant at the *p* = 0.1 significance level on univariate analysis were included in the multivariable model (age and sex of child, age and sex of guardian, skin problems, poor health, and previous hospital admissions).

b One missing value.

c 89 missing values.

d 86 missing values.

e 87 missing values.

f 87 missing values.

g Seven missing values.

h 16 missing values.

OR, odds ratio.

### Outcome of Provider-Initiated HIV Testing and Counselling

**Figure 1:**
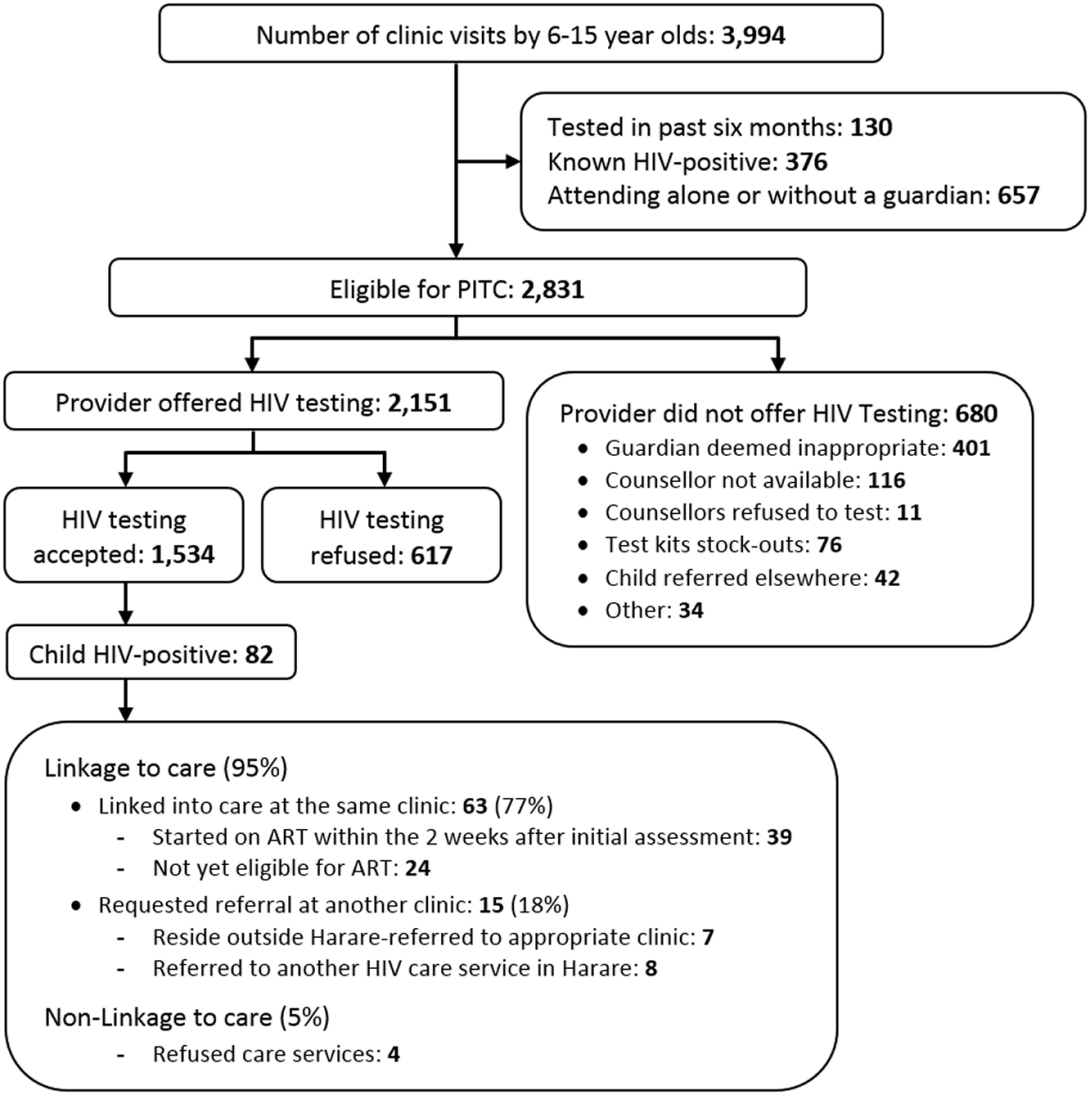
Study recruitment and outcome of provider-initiated HIV testing and counselling.

PITC was offered to 2,151 (76.0%) of the 2,831 eligible children (Figure 1). The reasons for not offering PITC were as follows: guardian deemed inappropriate to give consent by clinic staff (*n* = 401, 59.0%), counsellors not available (*n* = 116, 17.1%), stock-outs of testing kits (*n* = 76, 11.2%), counsellors refused to perform the test (*n* = 11, 1.6%), and referral to another health care facility (*n* = 42, 6.2%). Of the 2,151 children who were offered HIV testing, 164 (7.6%) did not assent, and 179 (8.3%) guardians declined consent for their child to be tested. The median age of children declining assent to testing was 10 y (IQR: 8–12 y). Ten mothers wanted to discuss with the child's father before consenting to the test. A further 264 (12.3%) children and guardians left the clinic before HIV testing could be performed, which may reflect an underlying unwillingness to test despite giving consent, or reluctance to wait for the testing procedure to be completed.

Children aged older than 11 y and children with a guardian less than 25 y of age or a male guardian had lower odds of being offered testing (Table 1). Children with previous hospital admissions, persistent skin problems, or poor health in the 3 mo prior to the visit were more likely to be offered HIV testing (Table 1). Orphans and children with self-reported poor health or with skin problems were significantly more likely to take up HIV testing (Table 2). Male guardians were more likely to decline consent for the child to be tested for HIV than female guardians (adjusted odds ratio 1.34; 95% CI 1.01–1.77).

**Table 2:** Association between baseline variables and child/guardian refusing HIV testing.

Category	Variables	All Children (*n* = 2,151)	HIV Testing Refused (*n* = 617)	HIV Testing Accepted (*n* = 1,534)	Univariate OR (95% CI)	Multivariable OR^a^ (95% CI)
**Child**	**Age in years, median (IQR)**	9 (7–11)	9 (7–11)	9 (7–11)		
	**Age by category, ** ***n*** ** (percent)**					
	≤7 y	608 (28.3%)	196 (31.8%)	412 (26.9%)	1	1
	8–11 y	1,045 (48.6%)	277 (44.9%)	768 (50.1%)	0.76 (0.61–0.94)	0.76 (0.60–0.96)
	>11 y	498 (23.2%)	144 (23.3%)	354 (23.1%)	0.86 (0.66–1.11)	0.93 (0.71–1.23)
	**Male, ** ***n*** ** (percent)** b	1167 (54.3%)	341 (55.3%)	826 (53.9%)	1.06 (0.88–1.28)	—
	**Orphan, ** ***n*** ** (percent)** c	297 (14.3%)	53 (9.7%)	244 (16.0%)	0.56 (0.41–0.77)	0.63 (0.45–0.87)
	**Previous hospital admission, ** ***n*** ** (percent)** d	213 (10.2%)	48 (8.7%)	165 (10.8%)	0.79 (0.57–1.11)	—
	**Poor health, ** ***n*** ** (percent)** e	155 (7.5%)	17 (3.1%)	138 (9.0%)	0.32 (0.19–0.54)	0.37 (0.22–0.63)
	**Skin problems, ** ***n*** ** (percent)** f	280 (13.5%)	56 (10.0%)	224 (14.7%)	0.66 (0.49–0.90)	0.73 (0.54–1.00)
**Guardian**	**Age in years, median (IQR)** g	34 (29–40)	34 (30–40)	34 (29–41)		
	**Age by category, ** ***n*** ** (percent)**					
	≤25 y	206 (9.6%)	52 (8.5%)	154 (10.1%)	0.78 (0.55–1.10)	—
	26–35 y	1,003 (46.8%)	303 (49.4%)	700 (45.8%)	1	—
	36–40 y	406 (18.9%)	129 (21.0%)	277 (18.1%)	1.08 (0.84–1.38)	—
	>40 y	529 (24.7%)	130 (21.2%)	399 (26.1%)	0.75 (0.59–0.96)	—
	**Male, ** ***n*** ** (percent)** h	295 (13.8%)	98 (16.0%)	197 (12.9%)	1.28 (0.98–1.67)	1.34 (1.01–1.77)

a *n* = 2,061 for the multivariable model. All variables significant at the *p* = 0.1 significance level on univariate analysis were included in the multivariable model (age of child, orphanhood, poor health, skin problems, and sex of guardian).

b One missing value.

c 73 missing values.

d 71 missing values.

e 72 missing values.

f 73 missing values.

g Seven missing values.

h 13 missing values.

OR, odds ratio.

Of the 2,831 children eligible for PITC, 1,534 (54.2%) underwent HIV testing, with 82 testing HIV-positive (HIV prevalence 5.3%; 95% CI 4.3%–6.6%). In addition, 940 (33.2%) accompanying guardians were also tested for HIV, of whom 186 (19.8%; 95% CI 17.3%–22.5%) tested HIV-positive.

The median age of children who tested HIV-positive was 11 y (IQR: 9–14 y), implying—for vertical transmission—a delay in HIV diagnosis of more than a decade. Overall, 78 (95%) children were linked to HIV care following diagnosis, of whom 63 (83%) chose to access care at the same clinic where they underwent HIV testing. More detailed information was available for these 63 children: 35 (55.5%) children were orphaned, of whom ten were paternal orphans, 18 maternal orphans, and seven double orphans. Fifty-eight children (92%) were thought to have acquired HIV vertically and three horizontally (aged 10, 10, and 14 y), and in two children parenteral transmission could not be definitively excluded. Overall, 58 (92.1%) had a missed opportunity for earlier diagnosis: 41 (65.1%) of the children had a parent or natural sibling who was already taking ART but the child had not been tested, two (3.2%) had been treated for tuberculosis, 19 (28.6%) had a past hospital admission, and 38 (60.3%) had attended a primary care clinic in the past 6 mo. Forty (63.5%) guardians had suspected that their child would test HIV-positive. The median CD4 count at diagnosis was 342 (IQR 244–522) cells/µl.

### Provider Perceptions and Experience of HIV Testing of Children

The interviews conducted with 12 clinic staff members illustrate the contextual factors associated with health care providers' decision-making around offering HIV testing to children.

#### Confusion about testing guidelines and regulations

HCWs demonstrated uncertainty about PITC regulations, particularly relating to consent procedures. They expressed confusion about the age at which a child could choose to test him/herself, what type of caregivers qualified as legal guardians, and whether guardians had to undergo testing themselves first. Most understood that children under the age of 16 y required parental permission, and believed that if a parent was not physically present but still alive, s/he would need to provide consent, regardless of who cared for the child on a daily basis. Children were often sent away to seek additional permissions, and frequently did not return. Most HCWs also believed testing of the guardian to be a mandatory, rather than recommended, step in the PITC process:

Very few come with their parents or legal guardians. That's where we face challenge. You will always need consent. Even if you see a sick child you have to encourage the person who came with the child to get consent. The child stays with the grandmother but comes with the aunt to the clinic. The aunt does not stay there....So that will create problems in cases of follow-ups and adherence. [female head nurse]

They will be afraid of being tested. They know we will not test the child unless it is in the best interest of the child without first testing caregiver. So they are too scared to bring the children because they do not want to know their status. [female counsellor]

For those whose parents are outside the country, some of them are not getting permission to be tested. We will then feel that our hands are tied. We will not be able to test the child without legal guidance. We will treat the child and ask the caregiver to go and talk to the parents of the child and come back. But they never come back. [female head nurse]

#### Concerns about PITC for children

Many HCWs expressed scepticism around the PITC initiative more generally, concerned that vulnerable children were tested without adequate counselling and provision of holistic “child-centred” services.

A child is different from an adult because she is vulnerable. I think that is what I can say about counselling a child, that it is difficult but we will now have a [large] workload....So we never have time to sit down with the child so that she really understands and to assess her understanding of what we are doing. We are just testing. [female counsellor]

Some HCWs did not offer testing for fear that children would be perceived as burdens in the household if they tested positive, leading to maltreatment, stigmatisation, or even abandonment.

Your worry is you test the child and the child will be abused. So most of the time we do not agree. [female counsellor]

#### Difficulties in implementation

HCWs complained that logistical arrangements were suboptimal for expanding PITC because of stock-outs of testing kits and long client waiting times. Increases in workload and insufficient space to provide comprehensive counselling were mentioned, and providers believed these conditions contributed to reasons why children left the clinic prior to testing, even if consent had been given:

For those who refuse it's because of lack of time. They were few counsellors to do the testing. The queue might bore them. They will just slip away. That's how they refuse most of the time. [female head nurse]

The other barrier that we have seen is space. You know this [counselling] requires privacy, [which] we do not have. [male head nurse]

## Discussion

This study showed a high prevalence of HIV infection amongst older children attending primary care services in Harare, Zimbabwe. The median CD4 count among children diagnosed was 342 cells/µl, significantly higher than that reported in another study of children that tested HIV-positive following admission to two hospitals in Harare (median CD4 count 145 cells/µl) [6], highlighting the benefits of PITC. In addition, a third of accompanying caregivers were also tested for HIV, with nearly 20% testing positive. HIV testing was offered to 76% of eligible attendees. This figure is likely to be higher than in routine settings because reasons for not offering HIV testing and for clients refusing consent to test were explicitly recorded.

Ninety-five percent of children who tested HIV-positive were linked to care, the majority choosing to access HIV care at the same clinic where they underwent HIV testing. Linkage to care rates in our study are substantially higher than those reported in other studies [13],[14]. This is likely to be a consequence of the availability of HIV care at the same clinic where the child tested. Studies have also shown that decentralised HIV care services may improve linkage to care by reducing the time and travel costs incurred by patients in accessing care at secondary level health facilities [15],[16].

More than 90% of children who tested HIV-positive had had previous contact with health services, had biological parents currently in HIV care, or had a verbal report of a previous positive test result but were not accessing HIV care. Previous studies have shown high rates of undiagnosed HIV among older children in sub-Saharan Africa, and it is therefore key that strategies for identifying older children living with HIV and linking them to HIV care are strengthened [5].

Overall, only 54% of eligible children underwent HIV testing; 19% of attendees were not eligible because they attended alone or with an unrelated individual, but nearly a quarter of those who were eligible were not offered HIV testing. From the information recorded for each untested child about why testing did not occur, the major reason that HCWs did not offer HIV testing was concern about the suitability of the accompanying caregiver to provide consent for testing. There is significant variation between African countries in policy regarding consent for HIV testing of unemancipated minors. The age at which children are deemed able to consent to HIV testing ranges from 12 to 21 y, with some countries allowing younger children to give consent independently if “mature” enough [17]–[19]. However, more than 30% of children in countries with severe HIV epidemics are AIDS orphans, and economic constraints cause many adults to migrate for work, leaving children under the care of extended families [20]. Consequently, a large proportion of children experience multiple and changing caregivers [21],[22]. Legal documentation of guardianship is uncommon, and the “type” of caregiver who is deemed suitable to give consent for a child to have an HIV test is not clearly defined in HIV testing guidelines [23]. Even where guidance is available within countries, national/international agencies providing HIV services may be unaware of local policies, and confusion amongst HCWs is compounded by the varied consent age and capacity requirements for health care interventions outside of the field of HIV care [19],[24].

Notably, the Zimbabwe national guidelines stipulate that in the absence of a caregiver, consent on behalf of the child can be obtained by a proxy authority such as a physician or social services, if testing is considered by HCWs to be in the “best interests of the child” [12]. In practice this is difficult to implement as primary care services are staffed primarily by nurses, and social services are often not readily accessible. This policy also places a heavy ethical responsibility on HCWs to decide what constitutes a child's “best interests”, and requires an understanding of the consequences of delayed treatment of HIV infection. An association was observed between HCWs offering testing and younger age of the child, previous hospital admissions, persistent skin problems, and poor health in recent months in the child. It is likely that HCWs may have been less stringent in labelling a guardian as “inappropriate”, or counsellors less likely to refuse to test, if the child appeared ill. Whilst this may be a form of “targeted” testing by HCWs, it may also reflect perceived “justification” of testing children who are unwell. In a seemingly well child, it may be less obvious that diagnosis of HIV as early as possible is in the best interests of the child. There is also limited knowledge of the risk of vertically acquired HIV infection in older children [25]. Children with longstanding HIV infection, even with relatively preserved CD4 counts, are at risk of organ damage and growth failure, and HCWs need to be educated about the benefits of HIV testing even in asymptomatic, older children [26]–[28].

A lack of counsellors and space were cited by HCWs as a reason for not offering testing. These resource constraints led to long client waiting times, resulting in many clients leaving the clinic before HIV testing could be carried out. Moreover, there were intermittent shortages of HIV testing kits, with testing prioritised for the programme for preventing mother-to-child transmission when stocks of testing kits were low, highlighting the verticality of HIV services and a perceived lack of importance of testing older children.

Availability of dedicated child-centred services was perceived as a requisite for PITC to be implemented, but may not be feasible in most resource-limited settings. HCWs felt they lacked the requisite skills to counsel guardians and children and to address HIV status disclosure to children. This has been identified as a barrier to HIV testing in other studies [29]. The lower probability of HCWs offering testing when the guardian was male or younger in this study may reflect a particular difficulty communicating with these groups. There is growing evidence of poorer access to and uptake of HIV services among men at every step of the HIV care cascade, and this is likely to have an impact on children accessing HIV testing when accompanied by male guardians [15],[30],[31]. In our study, the offer of HIV testing for the child was more likely to be refused if the child was accompanied by a male guardian.

The effectiveness of PITC for children relies not only on HCWs offering HIV testing, but also on guardians giving consent and children giving assent to be tested. Among clients who were offered HIV testing, 29% of guardians did not provide consent and 7.6% of children did not assent to testing. Lower rates of uptake of HIV testing by older children in other studies have been attributed to a lack of perceived personal risk of being infected, fear of the consequences of a positive result, and discomfort with PITC services [32]–[35]. While the benefits of HIV testing are widely recognised, studies have shown that there is limited awareness of the risk of older children having acquired HIV through vertical transmission [25]. The fear of stigma the child and the wider family may face is a disincentive for caregivers to have their children tested [34]–[36]. Caregivers' concerns that a child may not be old enough to understand and that they lack skills to discuss HIV with a younger child may explain why younger children were less likely to be tested [37]. Diagnosing a child with HIV infection is tantamount to disclosure of the parents' HIV status [36]–[38]. This fear may have been reinforced by the erroneous practice of health workers insisting that guardians themselves must be tested in order for their children to have an HIV test. This also raises an ethical issue about the parents' HIV status being revealed to the child and to the health care provider when the parent is alive but absent, and consent for the child to be tested is provided by a proxy caregiver. The HIV prevalence among children observed in this study may thus well be an underestimate, as guardians of children who were at risk of being HIV-infected may have been more reluctant to consent to testing for fear of their own HIV status being revealed [36].

To our knowledge, this is the first study investigating PITC provision in this age group in a public sector primary care setting. The strengths of the study are a large sample size and the application of quantitative as well as qualitative methods to understand the nuanced barriers to HIV testing in this age group. The study was conducted in routine health settings, making the findings generalisable.

The limitations are that we did not explore the reasons for refusal of HIV testing by clients. However, this has been the subject of other studies [25],[36],[38],[39]. The relationship of the child to the accompanying adult was not available, and thus the appropriateness of the guardian could not be judged. It was also not possible to investigate whether the type of relationship of the guardian to the child was associated with giving consent for the child to be tested, or with the concordance between the HIV status of the child and a biological parent. There was a higher proportion of missing observations for children not accepting HIV testing (12%) than for children who did accept HIV testing (1.2%). Finally, the availability of decentralised HIV care may have influenced the offering and uptake of HIV testing, although the impact of this is unlikely to be very significant as the availability of decentralised HIV care was not usually known to clients, and the numbers of children diagnosed with HIV and requiring onward care was relatively small.

Our study adds to existing literature showing a high prevalence of HIV amongst older children in sub-Saharan Africa, justifying the need for PITC in this population [40]. It highlights the missed opportunities for HIV testing of older children, and identifies opportunities to facilitate implementation of PITC for this age group (Box 1). Supply-side challenges are not insurmountable. Clear legislation concerning guardianship and consent needs to be introduced, and many examples of best practice do exist [23]. With improved clarity of guidelines, engagement with staff, and organisational adjustments within clinics, the commitment of HCWs can be harnessed to optimally implement PITC [41].

**Box 1. Strategies to Promote Provider-Initiated HIV Testing and Counselling in Children**

- Develop clear HIV testing policies and guidance regarding consent and guardianship:
  1. Who can provide consent for a child
  2. Age at which child can give independent consent
  3. Strategies to address cases of parental refusal for HIV testing of children
  4. Ethical guidance to address the inadvertent disclosure of absent parents' HIV status through a child's positive HIV test
- Provide legal authority to caregivers who are not parents or legal guardians to give proxy consent for medical care of a child
- Expand the role of designated HCWs to provide consent for the child if no parent or legal guardian is available
- Increase awareness among the general community and HCWs of the high prevalence of HIV infection among older children, even if asymptomatic, and education about benefits of prompt identification and treatment of HIV infection
- Train primary HCWs on counselling of children and guardians, maintenance of confidentiality, and regulations and policies about HIV testing of children and issues related to consent
- Address stigma and discrimination:
  1. Enactment of laws to protect HIV-infected children from discrimination
  2. Age-appropriate counselling to assist children and guardians facing stigma
  3. Development of initiatives to counter stigma at community level
- Address supply-side challenges:
  1. Streamlining the PITC process, e.g., through opt-out HIV testing
  2. Strengthening supply chain of HIV testing kits within health care facilities
  3. Task-shifting through use of lay counsellors for PITC
  4. On-site care plus linkages with other support services

## Abbreviations

ART: antiretroviral therapy
HCW: health care worker
IQR: interquartile range
PITC: provider-initiated HIV testing and counselling
